# The evolutionary ecology of molecular replicators

**DOI:** 10.1098/rsos.160235

**Published:** 2016-08-03

**Authors:** Sean Nee

**Affiliations:** The Braithwaite Group, Department of Ecosystem Science & Management, The Pennsylvania State University, University Park, PA 16802, USA

**Keywords:** sociomicrobiology, Price equation, mobilome, transposon evolution, virus evolution

## Abstract

By reasonable criteria, life on the Earth consists mainly of molecular replicators. These include viruses, transposons, transpovirons, coviruses and many more, with continuous new discoveries like Sputnik Virophage. Their study is inherently multidisciplinary, spanning microbiology, genetics, immunology and evolutionary theory, and the current view is that taking a unified approach has great power and promise. We support this with a new, unified, model of their evolutionary ecology, using contemporary evolutionary theory coupling the Price equation with game theory, studying the consequences of the molecular replicators' promiscuous use of each others' gene *products* for their natural history and evolutionary ecology. Even at this simple expository level, we can make a firm prediction of a new class of replicators exploiting viruses such as lentiviruses like SIVs, a family which includes HIV: these have been explicitly stated in the primary literature to be non-existent. Closely connected to this departure is the view that multicellular organism immunology is more about the management of chronic infections rather than the elimination of acute ones and new understandings emerging are changing our view of the kind of theatre we ourselves provide for the evolutionary play of molecular replicators. This study adds molecular replicators to bacteria in the emerging field of sociomicrobiology.

## 1. Introduction

The implications of promiscuous exchange of *information* for untangling the evolutionary history of molecular replicators, even between viruses and transposons inhabiting the three different domains of life, is currently under active review and development [1]. Here, we examine consequences of the promiscuous exploitation of the *products* of this information as part of the programme to develop a unified approach to the evolution of molecular replicators which has great promise [2,3].

First, we present a simple model for an evolutionary game played by molecular replicators which is based on the trade-off between investment in making (i) copies of the genetic information of the replicator and (ii) the machinery needed to do the copying. This is primarily an evolutionary model exploiting the fusion of evolutionary game theory—evolutionarily stable strategies (ESSs)—and the Price equation [4–6]. Then, we present an elaboration of the model to allow specialization, in which molecular replicators rely on each other for essential gene products. This is primarily an ecological model. Together, these constitute a new, unified model for use in understanding molecular replicators. Both sections will combine the presentation of theoretical ideas in conjunction with empirical generalizations. This treatment combination is needed because of the inherently multidisciplinary subject matter of the evolutionary ecology of molecular replicators, which spans the molecular biology of virology, genetics, immunology and the current statistical theories of evolutionary biology. Progress requires shared knowledge of facts, theory and vocabulary.

Looking at the larger picture leads here inexorably to the specific conclusion that an entire class of molecular replicators must exist, although the absence of evidence for their existence has been explicitly seen as evidence of their *non-*existence, requiring explanation. Our unequivocal view is that, in particular, molecular parasites of lentiviruses like SIV do exist, as yet undiscovered, and the reasons for this highlight the multidisciplinary nature of the study.

## 2. Evolution model

Molecular replicators comprise a template—a programme of information, such as the double-stranded DNA of herpes virus—and the machinery encoded by the templates for their replication—such as a virus-encoded DNA polymerase, a protein that copies the template. The parts from which templates and machinery are assembled are typically nucleotides and amino acids and combinations of these, and the production of new viral replicators has recently come to be described as occurring in virus ‘factories’ [7], an appropriate metaphor. The usual templates, replicating machinery and notable examples of such molecular replicators are given in table 1.

**Table 1.** Molecular replicators—common templates and replication machinery.

template	replication machinery	examples
ds DNA (double-stranded DNA)	DNA polymerase transposase reverse transcriptase miscellaneous	adenovirus, the common cold viruses transposons, the bulk of the eukaryotic genome
ss DNA (single-stranded DNA)	DNA polymerase miscellaneous	microviridae, viruses infecting bacteria that infect humans
ds RNA	RNA-dependent RNA polymerase (RdRp) miscellaneous	rotaviruses, cause gastroenteritis
+RNA (ss-positive sense RNA, can act as a messenger RNA)	RdRp reverse transcriptase miscellaneous	rhinoviruses, cause common coldHIV
‒RNA (ss-negative sense, cannot act as a messenger)	RdRp miscellaneous	influenza viruses
miscellaneous	miscellaneous	pleolipoviruses, viruses with both dsDNA- and ssDNA-infecting Archaea [8]

These molecular replicators may comprise just one-component machines, e.g. simple transposons known as Insertion Sequences [9] now known from all domains of Life, consisting of a DNA template coding for a replicating machine called a transposase. Or the machinery may have additional components: LINE-1 retrotransposons, which constitute about 20% of the human genome, provide many examples of replicators with two-component machines [10] and nodaviruses also provide many examples: one component is a capsid [11], a protein coat for travel between fish and fly hosts. The second protein ‘component’ has many functional domains, an RdRp domain, which has an obvious direct replicating function, and domains involved in other biochemical functions necessary for a healthy life. This illustrates the fact that replicating machinery may function directly, making copies of the templates, or indirectly, filling other fitness roles as wide ranging as movement within a host or protecting the host from other molecular replicators. Examples of the latter include antibiotic resistance genes which have been known and important since the dawn of molecular biology.

For this simple model, we will assume that there is a template for a machine with just one component (a replicase), but this is not a crucial assumption for our conclusions, so when we refer to a ‘replicase’ in the model, we mean any relevant component of the machinery.

The strategy of a focal template is *x*, the probability a copy of a template goes to become machinery or, with probability 1 − *x*, remains as an additional template, so when a template is copied, the copy may remain a template or go into pathways that produce machinery for replicating templates. The assumption that there is a choice between these pathways is a truth whose details are extraordinarily diverse and complex (e.g. [12]) with convenient summaries in theoretical work studying possible consequences of the trade-off for, for example, the ‘stamping machine’ model of poliovirus replication (e.g. [13]). Since replicators may use each others' information—gene—products, the machinery, the evolutionary optimization problem for strategy *x* is a game-theoretic one, as the fitness of a focal template, a player, will depend on the strategies of the others.

We suppose that, on average, there are *P*—for Pool—other players in a game apart from the focal template playing strategy *x*, e.g. active viruses in an infection or active transposons in a genome. It is very important to note the distinction between what we are calling a Pool, the players in a particular game, and the idea of the population at large, in which evolution occurs as a result of the outcomes of the games. A specific gaming analogy is this: the Pool is the players at a particular blackjack table, which may be surrounded by non-playing onlookers, and the population is all the players in Las Vegas. The numerical distinction between active players, *P*, and those just standing around the table can be stark: although around 17% of the human genome is LINE-1 transposons, only around 100 are active [10]. The importance of this distinction will become clear. To avoid multiplying subscripts, we will denote the average strategy of the other members of members of the Pool, distinct from the focal player, as *y*.

From the point of view of a focal replicator, a simple mass action expression of the copying of its template by a replicase is

2.1 (1−x)(x+Py).

As we are interested in generally valid insights, we do not construct a complex parametrized model for a specific biology. Instead, we construct a meaningful approximate model focusing on this simple game-theoretic mass action kernel to allow generalizations about the natural history of molecular replicators.

So, as a biological caricature, we write the fitness, *W*, of a focal template as proportional to

2.2 W(x,y)∝(1−x)(x+Py).

We now use the modern fusion of the Price equation approach and ESS analysis [4–6] which is ideally suited for understanding this model in the context of molecular replicators.

Basic calculus gives us the relationship between fitness of a focal replicator and its strategy as computed by

2.3 $$\frac{dW(x,y)}{dx}=\frac{\partial W(x,y)}{\partial x}\frac{dx}{dx}+\frac{\partial W(x,y)}{\partial y}\frac{dy}{dx}.$$

So far, this is nothing more than Darwin's approach based on the fitness of a focal individual, as a function of its strategy *x*. It explicitly includes the possibility that this personal fitness is affected by the strategies of the individuals involved in any game it may be playing, *y*.

We now move beyond Darwin to include the idea of evolutionary game theory, the ESS [14,15]. We want to find the strategy, *x*_ESS_ which has the property that if all the other replicators are playing it, *y* = *x*_ESS_, then the focal individual cannot have a higher fitness by playing a different strategy. With that idea, we wish to compute:

2.4 $$\frac{dW(x,y)}{dx}=0,\;\mathrm{when}\;x=y=x_{\mathrm{ESS}}.$$

Calculating this, in equation (2.3) we note that d*x*/d*x* = 1. The relationship between the average strategies of the others in the game pool, *y*, and the strategy of the focal individual, *x*, d*y*/d*x*, we now symbolize by *r*, yielding

2.5 $$x_{\mathrm{ESS}}=\frac{1+Pr}{2+P+Pr}.$$

The Price equation approach now manifests itself in the recognition of *r* as the statistical relationship between the average strategy of the Pool of players in the game a focal template finds itself and the strategy of the focal template itself. We can readily move between the calculus of d*y*/d*x*, with its intuitively clear idea of relationship, and the statistical concept of *r—*a relationship such as a correlation or a regression slope, appropriately scaled—and the subject has been given book-length treatment in the specific context of social evolution [4]. If there is no relationship between *y* and *x*, so there is independence, then *r* = 0 and we are back to Darwin unplugged.

Indeed, a simpler version of this result, equation (2.5), was first derived [16] in a model of the evolution of retroviruses and retrotransposons with *r* = 0. Lacking the tools of the contemporary theory of social evolution, that assumed that each game consisted of entirely random players, so d*y*/d*x* = 0. The transition from the assumption of complete independence is a qualitative one, not one of degree, and this requires contemporary tools. These, in turn, allow the development in understanding described in the rest of this paper.

We do not, in the general case, need to specify a particular model generating the statistical relationship, *r*, and we have not yet done so. And apart from the ESS idea, which Darwin lacked, we have simply equipped his understanding with modern statistical tools.

The contemporary theory of social evolution [4–6], which we have sketched with equations (2.3) and (2.4) and then substituting d*y*/d*x* = *r* brings simplicity and power resulting from its freedom from any particular biology, hence its usefulness for molecular replicators. A demonstration of this, explicitly contrasted with previous approaches, was provided by a study of the social problem of sex ratio evolution in the face of the bizarre and complex biology of reproduction, transmission and population structure of protozoa [17]. More abstractly, we can apply the del, ∇, operator analysis of equations (2.3) and (2.4) to any *W*(*x*, *y*) function, with *x*, *y* and *r* given the same interpretations as in this paper for a Frank–Taylor–Price social evolution analysis.

Social evolutionary biology has studied an enormous variety of models generating *r*, such as past experience [18], predator behaviour [19], reputation [20], etc., as well as genealogical relationships. Much confusion has arisen from the fact that quite distinct models may generate a statistical relationship between the strategy of a focal individual and the Pool in which it finds itself, i.e. between *x* and *y*. The fact that social evolution can be studied entirely from a personal fitness approach with *r* having an abstract, statistical meaning was perfectly understood by Hamilton, as evidenced by his study of the evolution of spite [21], which envisions *negative r*.

An example from daily life makes clear the intuitive, albeit abstract, nature of relationship, *r*. A coin has been tossed and comes up Heads: it is tossed a second time and comes up Heads again. The outcome is identical, but *r* = 0. By contrast, two instances of your signature are never, ever the same, but *r* = 1. More formally, each instance of your signature can be considered to consist of an identical, ‘ideal’ instance plus random noise.

To move forward, we now make the important point that *r* and *P* are not conceptually or evolutionarily conjoined. *P* varies between 0 and Infinity and refers to the number of active replicators. In this mass action model, equation (2.1), *r* varies between 0 and 1. The relationship *r* may be determined by outside agencies, such as predators in the case of the evolution of warning coloration [19], which, in a different general model, may generate a negative *r*. For example, in influenza virus, the capsid component of the machinery may choose to encapsidate template elements that are complementary.

Biologically, *P* may be affected in viruses, for example, by the size of an inoculum from a single infected individual and *r* by the number of vectors that contribute to an infection over its course. For transposons, *r* will be affected by the breeding system of the genomes in which the transposons are located and the recombination rate and numbers of transposons at various distances around their genome location. *P* and *r* may be connected in exotic ways if the activation of quiescent replicators is in any way a function of their strategies which may be the case for Insertion Sequences, for example.

## 3. Ecology model: specialization and division of labour

In this section, we are interested in molecular replicators that are dependent for their existence on the products of other molecular replicators. Table 2 provides a Bestiary of this new ecology.

**Table 2.** A Bestiary of molecular replicators.

examples	category	notes
satellite tobacco mosaic virus	satellite virus	a satellite *virus* encodes its own coat protein but relies on Helper for other machinery
CMV Y-sat, cucumber mosaic virus (CMV) Y satellite RNA	satellite RNAsubviral component	a satellite *nucleic acid* like RNA [22–24] uses Helper coat protein, may provide other non-protein machinery, e.g. siRNAs [25]
alphavirusesbetaviruses	satellite DNAsubviral components	nomenclature of newly discovered classes of replicators is in chaos, what needs/uses what/anything where/anywhere?
tobacco rattle virus comovirusesdianthoviruses	multi-component viruses, genome components separately encapsidated‘coviruses’, for short	contrast with influenza virus whose genome segments travel in same capsid original [26] shorter term, coviruses, almost [16] never usedvery common form of plant viruses
Sputnik [27]	satellite virusvirophage	virus of Mamavirus which infects amoebae
unnamed	transpovironsprovirophage	giant viruses such as mimivirus family and Pandoravirus are revealing a new bestiary of replicators [2]
sTRSV, tobacco ringspot virus	viroidviroid-likevirusoidsatellite	‘s’ prefix generically ‘satellite’, various names reflecting knowledge of relatedness, contribution of any sort to Helper virus, known requirements for components, etc. [11]
P4, Enterobacteria phage P4 [28]	satellite (bacterio)phage	depends on P2 phage
Hepatitis D virus	satellite RNAsubviral satelliteviroid-likemore?	see [29]uses coat protein of Hepatitis B virus
pea enation mosaic virus	?	insoluble ontological conundrum [30,31]
V-SINES	retropositional parasitesnon-autonomous transposable element	rather than being simply defective LINEs, V-SINEs may actively parasitize them [32]

We require a label to distinguish molecular replicators that will not occur in such a list from those that do. A molecular replicator like that causing the common cold does not occur here, because it relies entirely on replicating machinery that it encodes for itself or is encoded by the metazoan host. All those in table 2 require products encoded by other molecular replicators and the nomenclature is a mess. So we choose a label not in the table—DI replicators.

The acronym DI has been attached to ubiquitous phenomena that have been observed since molecular biology began. The phenomena come with a Babel of names reflecting the different disciplines that have observed them. One common compound word [33] is the prefix Defective Interfering, DI-, suffixed by -viruses, -particles, -RNA or -DNA. They are of particular interest here because they readily arise in the laboratory culture of every single virus where they have been looked for, with the curious exception of vaccinia virus, and can persist readily in laboratory culture, even to the extent of exhibiting long-term coevolutionary games with their progenitor [34] and pairs of mutually complementing DI viruses completely replacing the progenitor [35]. The DI replicators may compensate for their deficiencies with higher replication rates by being shorter [16] or less obvious means [36,37].

We do not want the repeated use of the word ‘deficiency’ to suggest we are restricting our attention to any particular ecological relationship. Quite the contrary and, as discussed at length elsewhere for the rich ecology of molecular replicators [30], how one views ecological relationships is flexible: is our relationship with domestic chickens predatory or mutualistic? our mitochondria? each other? etc.

The use of the word ‘interfering’ as a contributor to the DI acronym is important. DI viruses are identified as such in laboratory studies by their ability to replicate at the expense of the progenitor viruses whose products they parasitize. This has led to an interest in the possible use of them in treatment and prophylaxis [38]. They are not to be confused with the simply defective viruses of live attenuated vaccines or retroviruses that are defective having been manufactured for the purposes of gene therapy [39].

The use of DI replicator as a generic term for the naturally occurring phenomena of table 2 is justified because their deficiencies in one capacity are substituted by other replicators. This can be a logical necessity in systems such as viroids or virusoids which encode for nothing at all. It can be directly observed in many systems: for example, in the covirus tobacco rattle virus, the ‘long-particle’ containing the replicase template can establish successful infection within a plant but requires the ‘short-particle’ containing the template for the capsid to move between plants [30].

Most importantly, deficiency substitution can be inferred from the natural occurrence of DIs and not just their transient appearance. In these cases, the ‘deficiency’ has the same status as the deficiency of carnivory compared with omnivory, or the inability to photosynthesize is a ‘deficiency’ for many bacteria. This makes the DI acronym very useful for evolutionary ecology, as it could be applied to lions which are unable to photosynthesize as opposed to lions which are born without teeth, the latter being simply defective.

The first requirement for a replicator to engage in interesting ecological relationships is to forgo entirely the encoding of particular functions and rely entirely on the substitutable products of others. In the basic model, the strategy *x* for a focal replicator is only optimized arbitrarily close to *x* = 0 when the number of other players in the game, *P*, becomes infinitely large and the strategies they are following are statistically independent, *r* = 0. In words, there is no point in investing in machinery if all you want for replication is freely available.

So, now we start at the boundary *x* = 0, with the possibility that a template dispenses altogether with some or all of the information for machinery and acquires a replication advantage when its deficiency is complemented. We return to our simple model and model the conditions for a mutant playing *x* = 0, to invade a population of replicators playing the ESS strategy.

For the first time, we will adopt an explicit model for *r*, the statistical relationship between replicator strategies. This is a model in between a purely statistical one, which has a pure modelling framework in terms of mathematical probability theory, and a physical model specifying biological details.

Observing a focal replicator, with probability *r* we ‘pair’ it with a replicator with an identical strategy, and with probability 1 − *r* we pair it at random with a replicator from the population—world—at large. This model generates a relationship, *r*, which is mathematically the correlation coefficient. It has long been used in population genetics [40] although it was disliked by Sewall Wright [41] as it inclined one to an unnecessary obsession with positive correlations.

We suppose that initially a mutant replicator is so rare that its impact on the selective environment of the resident strategy can be ignored—from the point of view of the resident, the mutant does not exist. The fate of a *unique* mutant is determined by stochastic modelling quite different to what we are doing here. So too, are the stochastic processes associated with complementation, such as obviously arise with coviruses, when numbers are very small. A replicator whose dynamics are in the zone of stochasticity has the larger, ecological, problem of (i) existence at all and (ii) if its colonization/infection propagule size is so small as to make complementation of defectives problematic, then the persistence of the replicator is, again, itself ecologically problematic.

But whatever biological factors are affecting the selective environment of replicators in general, such as modes of transmission, affecting the statistical associations between the members of any particular game and thus determining the evolutionary outcome, these are also experienced by the rare mutant itself. This makes it meaningful to have the mutant experience the consequences of these factors in terms of the strategies it finds its own fitness affected by, in other words, to see the world from its own statistical perspective. Given this framework, we write the condition for the spread of a rare mutant producing only template as

3.1 $$(1-r)P\;x_{\mathrm{ESS}}\alpha >(1-x_{\mathrm{ESS}})(P+1)x_{\mathrm{ESS}},$$

where *α*, *α* > 1, is the advantage gained by the mutant, enjoyed when others make up its deficiencies. This gives us the size of the advantage a ‘defective’ replicator must enjoy if it is to establish in a world of complete replicators playing the ESS strategy, equation (2.5):

3.2 $$\alpha >\frac{{\left(P+1\right)}^{2}}{P(1-r)(2+P+Pr)}.$$

The advantage threshold always increases with increasing *r* and decreases with increasing *P*.

Note that we have kept the first and second models separate, rather than attempt to merge them into a more elegant supermodel in this paper, for several reasons, not least of which is to allow attention to focus easily on the different conceptual elements.

We will now describe the sorts of ‘worlds’ we expect to observe in the same way that one makes ecological generalizations about, for example, environments with different levels of productivity. We do this by making generalizations based on qualitative inference about the difficulty of invasion of DI replicators. The first three are not problematic.

## 4. Three straightforward ‘worlds’

### 4.1. Low-*P*/High-*r*

We recall that *P* refers to active replicators. A replicator in a Low-*P*/High-*r* world could be a virus that never multiplies to any appreciable level in its host and is a highly short-lived infection in hosts that rarely experience multiple infection, so *r* is High. Such a replicator might be of interest as a curio of epidemiology if it was ever discovered to exist, because its long-term persistence is an ecological problem of avoiding extinction if between-host transmission is always by a small number of propagules.

### 4.2. Low-*P*/Low-*r*

This may be particularly relevant for transposable elements. Mendelian recombination, outbreeding and assortment are very effective means of stirring the molecular statistical soup, generating Low-*r* and its consequent stimulus for game playing by lowering the threshold of DI invasion*.* But residence in a genome removes the ecological persistence problem faced by Low-*P* replicators, and an environment of Low-*P* replicators is not conducive to game playing. Given the opposite directions in which these factors operate, we cannot make generalizations from this level of modelling.

Although it is known that autonomous active transposons, the independently wealthy players at the blackjack tables, are a minute fraction of the totality [42], interest in all the others has been focused on their impact on host genome evolution, i.e. their impact on the casino. So, the framework presented here is certainly consistent with the apparently low level of game playing among these replicators, but this may simply reflect our personal interests. It is also possible that our genomes provide the equivalent of war cemeteries after the combatants have evolved themselves to mutual annihilation.

### 4.3. High-*P*/Low-*r*

This is the well-mixed soup where all the creative potential of molecular game playing can flourish as the invasion threshold for DI replicators is at its lowest. Everything we have discussed so far and seen in tables 1 and 2 thrives in this world which has been in existence for 3.5 billion years. Even the multi-cellular newcomers, around for just 650 million years, are fusions at both the cellular and genomic level [43], constructed from the ancient prokaryotic casinos whose creativity we are still just beginning to exploit with the likes of, for example, CRISPR [44]. The concept of mobilome [2] is just one of many unifying highlights now emerging from the exploration of this world.

## 5. High-*P*/High-*r* world: animal viruses?

A High-*r* lowers the invasion potential of DI replicators and a High-*P* removes the ecological problem of persistence. Therefore, in these circumstances, we would expect to see self-sufficient replicators interacting with the larger world around them and not involved in games with other molecular replicators such as we have been modelling, and whose natural history we have been reviewing in table 2 and elsewhere in the text.

Possible candidate members of this club are the animal viruses, particularly those which we are so intimately acquainted with, such as cold and flu viruses. Our interest in our own infectious acute diseases may have given us a peculiar view of the multicellular organism environment in which many molecular replicators thrive. Now we will explore the idea that these are misleading anomalies, hopelessly distorting our view of even the molecular replicators that call us home.

Animal viruses certainly enjoy High-*P* during active infection, but what about *r*? Two things matter. First, the effectiveness in raising *r* of the animal immune system both by rapidly eliminating infection and preventing superinfection. Second, the effect on *r* of bottlenecks in the infecting virus inoculum resulting in a relatively small *P* at the initial time of infection, which coupled with a lack of superinfection, would be naively thought to generate High-*r*. We may have greatly overestimated both these factors.

### 5.1. Theory

There are two well-established results from mathematical population genetics that have great relevance here. First, migration into a population is such a counterintuitively potent mixing force, reducing *r*, that it has been given its own acronym in conservation genetics where it is of particular interest—the OMPG rule, for One Migrant Per Generation [45]. Second, bottlenecks are ineffective in increasing *r* to a remarkably counterintuitive degree with implications in many areas [46]. We will look at these briefly in turn.

#### 5.1.1. The effects of immigration on *r*

There is a large, venerable literature in population genetics going back nearly a century, mainly to Sewall Wright, the conclusions of which can be summed up in the surprising idea that just one migrant replicator a generation is sufficient to maintain a Low-*r*, in our terms. This result has the intellectual status of the Hardy–Weinberg Equilibrium in that it is true and much of the subsequent development of the subject concerns efforts to describe caveats and conditions under which it is not true. Most of the literature has the domestication concerns of diploid Mendelian genetics, but a coalescence approach is freeing it from that [47], rendering it possible to discuss the subject in the general context of molecular replicators. This result is taken as the starting point of our own perspective on the evolution of molecular replicators as no other starting point presents itself.

#### 5.1.2. The effects of bottlenecks on *r*

The basic counterintuitive result is that even a large bottleneck, reducing *P* transiently in size, has little effect on *r*. Again, the coalescence approach frees this result (e.g. [46]) from all its baggage in the plant and animal origins of population genetics. Abstractly, the result is as follows. Consider the genealogy of any replicators—transposons, species, etc.—existing at a particular time and visualize them as the tips of a genealogical tree. Prune the trees—representing, for example, the generation of a new virus infection by a propagule consisting of the clippings from trees of infection in a single host, or a mass extinction throwing the clippings away. The amount of evolutionary history represented in the survivors, i.e. the total branch lengths of the genealogy connecting them, can be very large—simply visualize pruning a tree which leaves big branches behind, a history represented in both the clippings and the leaves that remain [46]. Mathematically, *r* can be computed for the survivors and it is largely unchanged, in accord with the visual intuition. This result is as valid for molecular replicators as anything else.

The message is clear: our starting point for the understanding of molecular replicator games must be the surprising, but theoretically well informed, view that the combination of bottlenecks and low levels of superinfection are weak forces for the maintenance of a High-P/High-*r* regime. So this is by no means a natural assumption even for animal viruses.

### 5.2. Biology

We are ignorant of both of the true extent and nature of chronic disease in even the human virome [48] and crop plants [49]. This reflects our natural interest in acute disease, rather than our coexistence with a microbiome that largely leaves us untroubled. But one emerging generality is clear. The goal of much immune response machinery is the management and control of infectious agents, not their elimination. In plant virus infections, limitation is confined to the several hundred cells of a lesion and resistance to superinfection by related strains is limited to areas around the lesions [50]; notable exceptions include plant-wide activation of RNA interference mechanisms [51]. In vertebrates, an entire arm of the immune response, Th2, may be concerned with the management of infection, not its elimination [52].

### 5.3. Superinfection

Our interest here does not concern the possibilities for recombination in the context of coevolutionary battles between host and replicator, such as the creation of new combinations of epitopes. Our interest is in the field it creates for evolutionary games between the replicators, the information for the strategies of which may be entirely invisible to direct recognition by immune systems. So, for example, the multiplication or deletion of replicase initiation sites is not looked for in investigations concerned with surface antigens. This is also relevant for superinfection exclusion, the phenomenon whereby partial superinfection protection is strain-specific [53].

The evidence suggests that superinfection is the norm for chronic viral infections. Military vaccine trial data exists showing that superinfection with HIV occurs effectively even immediately with sufficient frequency to allow inferences about disease progression [54]. In the longer term, superinfection is common [55] in HIV and SIV [56] and incidence is reported to be 40% in a large long-term study [57]. It is commonly observed in healthy individuals with Epstein–Barr virus [58,59] and cytomegalovirus [60] and *in vitro* in alphaherpesviruses [61]. Note that we are intentionally using the word ‘chronic’ and are not discussing persistent infections which may exist in a dormant, Low-*P*, state.

All theory and data lead inescapably to the view that chronic viruses should exhibit DI phenomena. But the absence of evidence is becoming taken as the evidence of non-existence of DIs, in particular of lentiviruses like SIV viruses, mainly because these viruses have been extensively sequenced and, yet, not revealed any [37]. A more compelling new argument against their existence is presented, and rejected, in the Concluding remarks. But here we reject the sequence argument because, historically, sequence searches come into play *after* we know what to look for, a task that is challenging and hugely rewarding, e.g. the metagenomics of the virome [62–64]. Even the initial discovery of a new retrovirus, HIV, did not involve sequencing: it was discovered as a new virus by a combination of observation (morphology), weighing (sedimentation), reverse transcriptase activity and serology. Even though sequencing technology is relatively recent, it is striking that both historically, and today, major DI discoveries are made not by sequencing, although once discovered their study is certainly furthered by it.

All the DI replicators in table 2 were discovered in ways that seem quaint by the standards of 2016. Satellite viruses and satellite RNAs of plants [23,24] were discovered visually, by weight (sediment bands) and serologically [65–67]. Coviruses of plants were discovered by their unusual infection kinetics [68] as well as these ways. More recently, such modern replicators as satellite virophage, Sputnik [27] have been first discovered visually. Hepatitis D was nearly completely overlooked and ultimately discovered by a Byzantine process sprinkled with serendipity that evades simple description [69].

So, we nearly lack even one example, Hepatitis D, of a natural human DI virus, but are not surprised by its existence given that hepatitis viruses have clearly been evolving in humans for a long time in spite of the confusion reigning in studies of molecular phylogenies [70]. We lack a theoretical basis to make firm statements regarding recent zoonoses like HIV simply because it is not clear if it has had time to evolve in humans as we expect, or to acquire other ecological partners by a different route. But by combining contemporary social evolution theory with our growing knowledge of virus biology, we can predict the existence of DI replicators in SIVs: these viruses are ancient, chronic and the primate immune systems have adapted to managing them in a chronic state of High-*P* [71]*.* It would be truly astonishing if they did not exist. Indeed, given the clear propensity of HIV to superinfection, it would be unsurprising if it had been accompanied with a fellow traveller when the zoonosis occurred.

We can go further. Agriculture has been of intense interest to us for 10 000 years and plant virus systems have the feature that infection is obvious, localized, locally abundant, easily extracted and cultivated, so it is not surprising that so many discoveries of various kinds of DI replicators have been made in plant systems. We see no reason for the same diversity not to exist in animals, waiting for means, motive and opportunity to unveil it.

## 6. Concluding remarks

Our prediction of a new class of DI replicators is made bold by referring specifically to retroviruses like SIVs, rather than just chronic viruses of ‘some sort’. This is because there are no examples of retroviruses of any kind in the ecological Bestiary of table 2. So perhaps there is just something about retrovirus molecular biology that rules out these games. It will not do to give the obvious answer that plant retroviruses have not been identified and plants, and now amoebae, are the main arena for the discovery of these players. This answer is flawed because plant genomes, like ours, are packed with retrotransposons, raising the compelling question of, where are the DI retrotransposons? Close inspection reveals that the V-SINEs of table 2 may be a reach. The answer we prefer is the one wholly consistent with the framework presented here—all retrotransposons are known to live in a low-*P* world [42] which is not conducive to game playing. So, we reaffirm our prediction of the existence of DI SIVs.

The general framework that is emerging sees the High-*P*/Low-*r* world as the natural one for ‘free-living’ molecular replicators and the Low-*P*/Low-*r* world as inhabited by, probably, many replicators inhabiting genomes. The High-*P*/High-*r* world, occupied by a few animal viruses such as the viruses causing colds, is anomalous in the universe of molecular replicators and preoccupies us only for personal, not intellectual, reasons. On this view, the coviruses of plants and amoeba-dwelling entities like Sputnik Virophage, for example, are the norm. Accepting this allows the study of the evolution of molecular replicators to proceed unencumbered by the taxonomic affiliations of the environments they may be gaming in, to address more challenging phenomena like limitations on the numbers of covirus partners [36], threshold phenomena and ‘catastrophes’ in the evolution of coviruses [30] and difficulties in the design of HIV DIs [37].

Finally, we see this work as squarely fitting into the emerging field known as sociomicrobiology, which has enjoyed increasing success in fusing contemporary social evolution theory with expanding knowledge of the biological behavioural repertoire of bacteria [72]. The analysis presented in this paper simply includes even more minute entities into this discipline and the title could refer to sociomicrobiology instead of behavioural ecology, the latter simply coined to free the study of sociobiology from its political contaminants [73].

Another vista opening for sociomicrobiology, with the inclusion of molecular replicators into its purview, is the impact of these evolutionary ecological relationships on the host in which they may be found, quite apart from any coevolution between the hosts and these replicators.

## Data accessibility

There are no new data arising from this research.
